# Deuterium Incorporation Protects Cells from Oxidative Damage

**DOI:** 10.1155/2019/6528106

**Published:** 2019-07-18

**Authors:** Piero Sestili, Maurizio Brigotti, Cinzia Calcabrini, Eleonora Turrini, Valentina Arfilli, Domenica Carnicelli, Marco Lucarini, Andrea Mazzanti, Andrea Milelli, Valeria Righi, Carmela Fimognari

**Affiliations:** ^1^Department of Biomolecular Sciences, University of Urbino Carlo Bo, Urbino, Italy; ^2^Department of Experimental, Diagnostic and Specialty Medicine, Alma Mater Studiorum-University of Bologna, Bologna, Italy; ^3^Department for Life Quality Studies, Alma Mater Studiorum-University of Bologna, Rimini, Italy; ^4^Department of Chemistry “G. Ciamician”, Alma Mater Studiorum-University of Bologna, Bologna, Italy; ^5^Department of Industrial Chemistry “Toso Montanari”, Alma Mater Studiorum-University of Bologna, Bologna, Italy

## Abstract

In the cold environments of the interstellar medium, a variety of molecules in which a hydrogen (H) atom has been replaced by its heavier isotope deuterium (D) can be found. From its emergence, life had to counteract the toxic action of many agents, which posed a constant threat to its development and propagation. Oxygen-reactive species are archaic toxicants that lead to protein damage and genomic instability. Most of the oxidative lesions involve cleavage of C-H bonds and H abstraction. According to free radical chemistry principles, the substitution of D for H in oxidation-sensitive positions of cellular components should confer protection against the oxidative attack without compromising the chemical identity of the compounds. Here, we show that deuterated nucleosides and proteins protect from oxidative damage. Our data suggest a new, subtle but likely role of D in terrestrial life's evolution in that its inclusion in critical biomolecules might have facilitated their resistance during the infinite generations of life entities, cells, and organisms.

## 1. Introduction

Deuterium (D) enrichment occurs in interstellar medium. In these cold environments, a variety of molecules in which a hydrogen (H) atom has been replaced by its heavier isotope D can be found. Astonishingly, the abundance of deuterated molecules in interstellar clouds is by far more than one would expect by the overall cosmic environmental D/H ratio [1]. Comets and asteroids collected and delivered these high amounts of deuterated species within the solar system. For decades, an outstanding question has been the origin of the volatile compounds of Earth, especially water. Indeed, our planet formed at such high temperatures that any original water must have evaporated. Yet today, two-thirds of the surface is covered in water and this should have been delivered from space after Earth cooled down [2]. Recent data confirm that bulk Earth water, due to its fairly high D/H ratio, might be the result of the progressive local accretion or impacts of asteroids or comets containing water and deuterated water. Along with water, about 25 deuterated molecules, including methanol, have been found to date in the interstellar medium and delivered to Earth surface with the same mechanism [3, 4]. For these reasons, Earth biosphere D/H ratio is higher than that of the overall cosmic environment. It is now generally accepted that life first evolved around 3.5 billion years ago. From that time, life had to counteract the toxic action of environmental as well as of endogenous agents: UV light, cosmic rays, ionizing radiation, radiation bursts, and radical species, which posed a constant threat to its early development and propagation. Oxidative stress may arise from many conditions including the above ones, which are likely to have paralleled the history of life, from its beginning up to now. In this light, oxygen-reactive species can be considered as archaic toxicants.

Oxidative damage is one of the most frequent events leading to DNA lesions and contributing to genomic instability [5]. Specific oxidative DNA lesions have been identified so far. Among them, 8-hydroxy 2′-deoxyguanosine (8-OH-dG) represents the most studied oxidative DNA modification among at least 20 different species identified to date [6]. Another common product of oxidative damage to DNA bases are thymidine glycols [7], which, although considered weakly genotoxic and mutagenic lesions, are thought to be a cause of formation of C-T transition mutation. Clustered and tandem lesions [8]—whose biochemical effects are still being investigated—are thought to exert significant detrimental effects on DNA repair and replication, and to be much more dangerous than isolated lesions. Proteins are another sensitive target for oxidative attack. Damage to proteins exposed to oxidative stress has been studied since, at cellular level, they are the most concentrated macromolecular target (per 10^12^ leukocytes: 100 g protein, 6.9 g DNA, 8.2 g RNA, 15.6 g total lipid, and 2 g cholesterol) [9]. Oxidative lesions in proteins usually result in the cleavage of the backbone or the alteration of the side chains with formation of carbonyl derivatives [10].

Most of the oxidative lesions described above involve cleavage of C-H bonds and H abstraction. The vibration frequency of the chemical bond linking the two atoms is determined by their masses. For atoms with the same proton configuration (i.e., the same chemical element) but different masses (i.e., different isotopes), the vibration parameters will be different and will differently affect the bond cleavage rate: in other words, stronger bonds are formed by heavier isotopes. According to well-established free radical chemistry principles [11], it is conceivable that substitution of D for H in oxidation-sensitive positions of many cellular components that undergo irreversible chemical transformations, such as oxidation or nitration, should confer protection against the oxidative attack without compromising the chemical identity of the compounds. While this D isotope effect in H transfer reaction has been proven to occur in many atom H abstraction reactions, evidence that this effect is preserved “in vivo” has never been reported in the literature. Going back to the relatively high abundance of D in Earth biosphere, here we addressed the question of whether the inclusion of increasing amounts of basic macromolecule precursors containing D in specific sites (nucleic acids and proteins) might affect the resistance of cells to oxidative stress as an archaic toxic model: in other words, did and does D facilitate or inhibit life? Here, we show that deuterated nucleosides (D-nucleosides) and amino acids (D-AAs) protect from oxidative damage.

## 2. Materials and Methods

### 2.1. Deuterated Nucleosides and Proteins

[8-D]2′-Deoxyadenosine (D-dA), [2′,2′′-D_2_]2′-deoxyguanosine (D-dG), and [methyl-D_3_]thymidine (D-dT) were purchased from Omicron Biochemicals Inc. L-Arginine-3,3,4,4,5,5,6-D_7_ (D-Arg), L-lysine-3,3,4,4,5,5,6,6-D_8_ (D-Lys), and L-proline-2,5,5-D_3_ (D-Pro) were purchased from Medical Isotopes, Inc. D- and non-D-nucleosides were dissolved in water. D-AA and non-D-AAs were dissolved in water at 50 mM concentration.

### 2.2. Cell Cultures

Human Jurkat T leukemia cells (Istituto Zooprofilattico di Brescia) were suspended and cultured at 37°C with 5% CO_2_ in RPMI 1640 supplemented with 10% heat-inactivated bovine serum, 1% penicillin/streptomycin solution, and 1% L-glutamine solution (all obtained from Sigma-Aldrich).

### 2.3. Cell Viability

Cells were treated with different concentrations (0-12.5 mM) of D-nucleosides, non-D-nucleosides or with D-AA (1-6 mM) for 24 h (i.e., one cell cycle). The viability of Jurkat cells was analysed by Guava ViaCount Reagent (Merck Millipore), containing 7-amino-actinomycin (7-AAD) [12]. Cells were incubated with the Guava ViaCount Reagent in the dark for 5 min and then analysed by flow cytometry (easyCyte 5HT flow cytometer, Guava Technologies, Merck Millipore). Moreover, we used Guava Nexin Reagent (Merck Millipore), containing 7-AAD and annexin V-phycoerythrin, to distinguish the mechanism of cell death (i.e., apoptosis or necrosis) [13]. Cells were incubated with Guava Nexin Reagent for 20 min at room temperature in the dark before flow cytometric analysis. We calculated the IC_50_ (the concentration of D-nucleosides inducing cell death by 50% following one cell-cycle exposure) and used concentrations of ≤IC_50_ in the following experiments.

### 2.4. Determination of Deuterated Nucleosides Incorporated into the DNA of Jurkat Cells

Jurkat cells (4.5 × 10^6^/3 ml) were treated with 500 *μ*M for D-dA or dA or 100 *μ*M for D-dT or dT for 24 h. Cells were collected by centrifugation for 7 min at 130 *g*, washed in PBS, and their DNA was extracted with QIAamp DNA Mini Kit (QIAGEN), according to the manufacturer's instructions. DNA extracted was sublimated under vacuum in Savant Speed VAC SC110 Concentrator Centrifuge (Savant) to remove ethanol contamination, quantified by Tecan Infinite f200 PRO spectrophotometer (Tecan), and digested according to Quinlivan and Gregory [14]. Briefly, 40 *μ*g of DNA/2 ml H_2_O was treated with 100 U benzonase (DBA Italy s.r.l., Milan, Italy), 120 mU phosphodiesterase I (DBA Italy s.r.l.), and 80 U alkaline phosphatase (Sigma-Aldrich) in 2 ml Tris-HCl buffer (20 mM Tris-HCl, 100 mM NaCl, 20 mM MgCl_2_, pH 7.9; all obtained from Sigma-Aldrich) o.n. at 37°C. The resulting deoxynucleosides were subsequently analysed in NMR as described below.

### 2.5. NMR Experiments

The ^2^H spectra were recorded on a 600 MHz spectrometer using a broadband direct probe tuned to the ^2^H frequency and removing the cable of the lock signal of the spectrometer to avoid interferences. A dummy sample of water was used to monitor the stability of the signal (HDO at natural abundance), which proved to be stable up to 48000 scans (line width of <1.5 Hz, about 40 h acquisition time). The samples were prepared using Milli-Q water, and the natural abundance HDO signal was used as the internal standard for chemical shift calibration. The spectra were recorded in the FT mode with 16000 to 24000 scans, using a 10 kHz spectral window, 1.0 s acquisition time, and 2.0 s relaxation delay. The FID were zero-filled to 65k data points, and a line-broadening function (3 Hz) was applied before transformation. The standard samples were prepared with pure D-AA to determine the detection limit of the experiments.

The ^1^H NMR experiments were performed on Bruker AVANCE III HD 600 MHz spectrometer with an 5 mm liquid probe. The sample was prepared directly into 5 mm NMR tubes: frozen samples were thawed at room temperature and 600 *μ*l of sample was added with 100 *μ*l of D_2_O. All NMR spectra were acquired at 600.13 MHz, temperature of 298 K. The spectra were referenced to the adenosine signal at 8.24 ppm. For each sample, the ^1^H NMR spectrum was acquired with a composite pulse sequence (zgcppr) [15], with 5.0 s water-presaturation during relaxation delay, 20.02 kHz spectral width, 32k data points, 2k scans, and 2 dummy scans; the free induction decay (FID) was zero-filled once and Fourier-transformed with a line broadening of 0.5. Moreover, baseline correction was applied using a polynomial order 3.

### 2.6. DNA Damage

To allow incorporation into genomic DNA, Jurkat cells were incubated for 24 h with D-nucleotides; following a further 4 h of incubation in a D-nucleotide-free medium, cells were treated for 30 min at 37°C with H_2_O_2_. In some experiments, incubations with D-nucleosides were prolonged up to 36 or 48 h. Immediately after damage induction or at specific time intervals of repair, the samples were analysed by the fast-halo assay (FHA) [16, 17]. After the treatments, 4.0 × 10^4^ cells were resuspended in 0.1 ml ice-cold phosphate-buffered saline (8 g/l NaCl, 1.15 g/l Na_2_HPO_4_, 0.2 g/l KH_2_PO_4_, 0.2 g/l KCl) spiked with 5 mM ethylenediaminetetraacetic acid (EDTA), diluted with an equal volume of 1.0% low-melting agarose in PBS, sandwiched between an agarose-coated slide and a coverslip and left on ice to allow the gelification. After removing the coverslips, the slides were processed according to FHA or comet assay. For FHA, the slides were immersed in a pre-chilled alkaline buffer (NaOH 0.3 M, 1 mM EDTA) and incubated for 15 min on ice. Ethidium bromide (EB; final concentration: 10 *μ*g/ml) was added during the last 5 min of incubation. The slides were then destained for 5 min in distilled water. The comet assay was performed as described previously [18], with slight modifications [19]. The slides were immersed in ice-cold lysing solution buffered at pH 10.00 (2.5 M NaCl, 100 mM EDTA, 10 mM Tris, 1% sarkosyl, 5% dimethyl sulfoxide and 1% Triton X100) for 60 min, placed in a pre-chilled alkaline buffer (0.3 M NaOH and 1 mM EDTA, pH 13.5), and electrophoresed at 300 mA for 20 min at 14°C. Once electrophoresed, the slides were stained for 5 min with 10 *μ*g/ml EB and destained in distilled water. Nuclei were visualized using a Leica DMLB/DFC300F fluorescence microscope (Leica Microsystems); digitally acquired images were analysed with the ImageJ software. Results obtained with FHA were confirmed in selected experiments using the comet assay [18]. The extent of DNA strand scission is expressed as nuclear diffusion factor (NDF), which is the ratio between the total area of the halo plus nucleus and that of the nucleus (FHA), or the ratio between the total area of comet tail and nucleus and that of the nucleus (comet assay).

### 2.7. Protein Synthesis in Cell-Free System

Cell-free protein synthesis was measured by both rabbit reticulocyte lysate translating endogenous messengers (radioactive assay) and rabbit reticulocyte lysate translating *Renilla reniformis* luciferase mRNA (luminometric assay). Cell-free protein synthesis by rabbit reticulocyte lysate [20] translating endogenous messengers was measured as incorporation of [^3^H]leucine into proteins. Cell-free protein synthesis by rabbit reticulocyte lysate translating *R. reniformis* luciferase mRNA was measured by a luminometric assay with some modifications [20]. Jurkat cells' protein synthesis was evaluated in complete medium in the presence of [^3^H]leucine. Rabbit reticulocyte lysate was prepared as described in Penzo et al. [20] and used for the in vitro translation of endogenous mRNA, as previously described [21]. Standard reaction mixture (62.5 *μ*l) contained 30 mM HEPES-KOH, pH 7.5, 80 mM KCl, 1 mM magnesium acetate, 50 *μ*M of each AA except leucine, 1 mM ATP, 0.25 mM GTP, 5 mM creatine phosphate, 0.18 mg/ml creatine phosphokinase, 0.5 mM dithiothreitol, 0.4 mM spermidine, 0.24 *μ*M (1 *μ*Ci) [^3^H]leucine, and 25 *μ*l of lysate. To study the effect of D-AA on cell-free protein synthesis, the reaction mixtures were modified (i) by adding different concentrations of D-AA; and (ii) by substituting a single D-AA for the normal counterpart at the same 50 *μ*M concentration. We quantified the protein synthesis using the rate of incorporation of labelled leucine. After 7 min incubation at 28°C, 1 ml of 0.1 M KOH was added. The solution was decolourized with two drops of 35% (*w*/*v*) H_2_O_2_, and 1 ml of 20% (*w*/*v*) trichloroacetic acid (TCA) was added. The protein precipitate was collected on a Whatman GF/C filter and its radioactivity quantified through scintillation counting. Under these conditions, the [^3^H]leucine incorporated was 172,483 ± 36,721 dpm (mean ± standard deviation; *n* = 4). In some experiments, gel-filtered rabbit reticulocyte lysate poor in free AAs has been used [22]; in this case, the [^3^H]leucine incorporated was 140,509 ± 7,386 dpm (mean ± standard deviation; *n* = 3).

For cell-free protein synthesis by rabbit reticulocyte lysate translating *R. reniformis* luciferase mRNA (luminometric assay), capped mRNA encoding for RLuc (*Renilla reniformis* luciferase) was transcribed from BamHI-linearized pRL-CMV (Promega) using the mMessage mMachine T7 kit (Ambion), according to the manufacturer's protocol. Rabbit reticulocyte lysate was gel-filtered and supplemented with 25 *μ*M haemin. Translation *in vitro* was performed in 22 *μ*l reaction mixtures under the conditions described above with the following additions—5 mM AMPc, 0.2 mM glucose 6-phosphate, and 0.3 *μ*g *R. reniformis* luciferase mRNA, and modifications—concentrations of DTT and GTP were raised to 1 mM and 2.25 mM, respectively. The complete mixture was incubated at 28°C for 90 min, and the luciferase activity was measured according to Dual-Luciferase® reporter assay system (Promega) instructions.

### 2.8. Determination of Protein Synthesis in Whole Cells

The efficiency of translation in Jurkat cells (0.75 × 10^6^/500 *μ*l) treated with D-AA at the indicated concentrations for 24 h was measured as the rate of incorporation of labelled leucine during a 30 min incubation in complete medium containing trace amounts of [^3^H]leucine. This procedure has been described in detail elsewhere [23]. Under these conditions, the [^3^H]leucine incorporated by control cells was 28982 ± 4753 dpm (mean ± standard deviation; *n* = 3).

### 2.9. Determination by NMR of Free or Protein-Derived D-AA in Jurkat Cells

**Figure 1:**
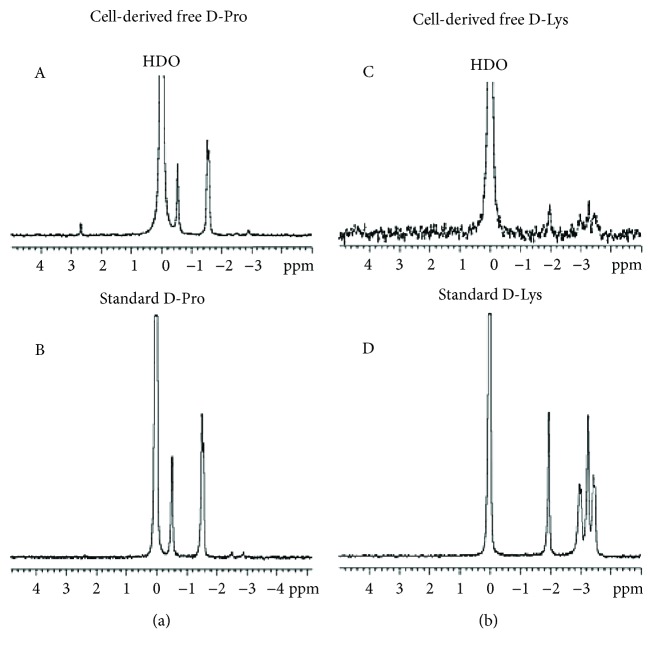
D-AA ^2^H-NMR spectra. (a and b) The ^2^H-NMR spectra of D-Pro and D-Lys, respectively. In A and C, TCA-soluble D-AAs were obtained after precipitation of cytosolic proteins from Jurkat cells treated with 5 mM D-AA. In B and D, true D-AAs (1 mM) were run under the same acidic conditions.

**Figure 2:**
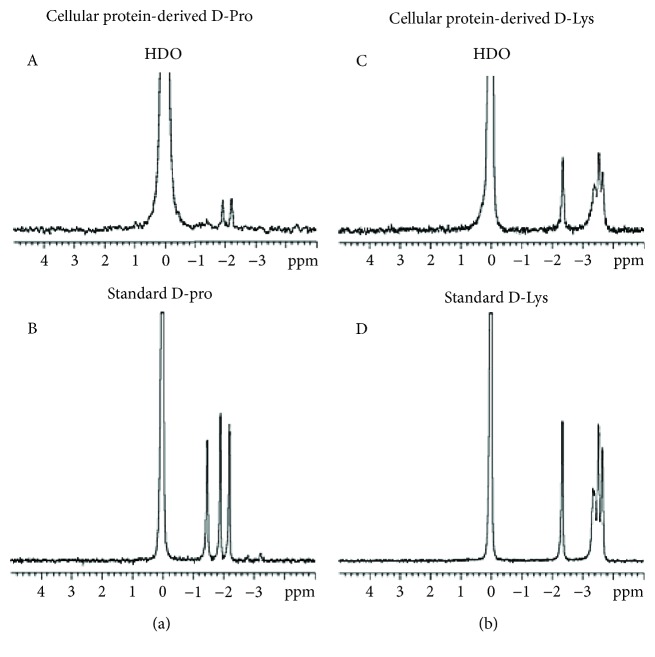
D-AA ^2^H-NMR spectra. (a and b) The ^2^H-NMR spectra of D-Pro and D-Lys, respectively. In A and C, D-AAs were obtained after alkaline hydrolysis of the precipitated cytosolic proteins from Jurkat cells treated with 5 mM D-AA. In B and D, true D-AAs (1 mM) were run under the same alkaline conditions.

Jurkat cells (9 × 10^6^/6 ml) were treated with 5 mM D-Pro and D-Lys as described above, centrifuged for 5 min at 200 *g*, washed three times with cold PBS, resuspended in 500 *μ*l of water, and sonicated in ice with a 3.2 mm diameter tip for 5 min at 95 W (15 sec pulse followed by 15 sec pause). The cellular proteins present in the postmitochondrial supernatant (prepared as described in the following section) were precipitated by addition of 100% (*w*/*v*) TCA to 10% final concentration. After centrifugation for 10 min at 13,000 *g*, the supernatants containing TCA-soluble free AAs were assayed by NMR. TCA-insoluble fractions were resuspended in 500 *μ*l 2N NaOH and incubated in sealed tubes for 24 h at 100°C [24]. The D-AA present in the alkaline hydrolysed were analysed by NMR. Each determination included a standard of D-AA in 10% TCA (Figure 1) or 2N NaOH (Figure 2). The amount of D-Pro or of D-Lys incorporated in cellular proteins was calculated from the standards obtained with known concentrations of the D-AA (1 mM in 2N NaOH). The results were expressed as nmol of D-AA/mg of cellular protein. The total protein content of each sample was measured by the Bradford assay [25].

### 2.10. Determination of Protein Carbonyls and Advanced Oxidation Protein Products in Jurkat Cells

Jurkat cells (3 × 10^6^/2 ml) were preincubated in the absence and in the presence of D-Pro, as described above. Cells were transferred in Eppendorf tubes, centrifuged 5 min at 200 *g*, and either untreated or treated with 5 mM H_2_O_2_ in PBS for 2 h at 37°C. At the end of the incubation, cells were pelleted as described above, washed twice with PBS, and lysed by adding 2 volumes of 50 mM Tris-HCl, 10 mM EDTA, and 1 mM phenylmethylsulfonyl fluoride [26]. After 10 min on ice, the suspension was frozen (15 min at −80°C) and, upon thawing, centrifuged for 10 min at 500 *g* at room temperature. Supernatant was centrifuged at 16,000 *g* for 10 min at 4°C, thus obtaining the postmitochondrial fraction. Protein carbonyls were measured on this fraction by using the method of Reznick and Packer [27] after precipitation of nucleic acids with 1% streptomycin sulfate (15 min on ice) [28]. Nucleic acids were discharged by centrifuging for 10 min at 6000 *g* at 4°C. The supernatants were treated with 4 volumes of 10 mM 2,4-dinitrophenylhydrazine in HCl 2.5 N for 1 h at room temperature in a rotator. The reacted proteins were precipitated by adding an equal volume of 20% TCA (10 min on ice). The final precipitate was obtained after centrifugation for 5 min at 13,000 *g*, washed three times with ethyl acetate/ethanol (1 : 1 *v*/*v*), and dissolved in 6 M guanidine hydrochloride. The absorbance of the sample was measured at 370 nm. The carbonyl content was calculated based on the molar extinction coefficient of 2,4-dinitrophenylhydrazine (2.2 × 10^4^ cm^−1^ M^−1^) and expressed as nmol/mg protein. Protein concentration was determined on aliquots of the same sample by the absorbance at 276 nm upon subtraction of the contribution of DNPH, i.e., A_276_–0.43 × A_370_, which gives protein concentration in mg/ml [29].

Levels of advanced oxidation protein products were determined according to the method of Kayali et al. [30]. Briefly, 5 *μ*l of the cell extracts obtained as described above was added to 70 *μ*l PBS and the A_280_ recorded. Then, 10 *μ*l of potassium iodide 1.16 M was added to the sample followed 2 min later by 20 *μ*l of acetic acid. The absorbance of the reaction mixture was immediately recorded at 340 nm. The concentration of the advanced oxidation protein products for each sample was calculated using the extinction coefficient 261 cm^−1^ M^−1^. The results are expressed as nmol/mg protein. Protein concentration was determined by the absorbance at 280 nm.

### 2.11. Statistical Analysis

Statistical analysis (GraphPad 5.0 software) was performed by Student's *t*-test (two-tailed), one-way, or two-way ANOVA with Bonferroni posttest analysis, as appropriate. A value of *P* < 0.05 was considered statistically significant. Graphs present the mean value of standard error of the mean (SEM) or standard deviation.

## 3. Results and Discussion

### 3.1. D-Nucleosides Protect from Oxidative DNA Damage

**Scheme 1:**
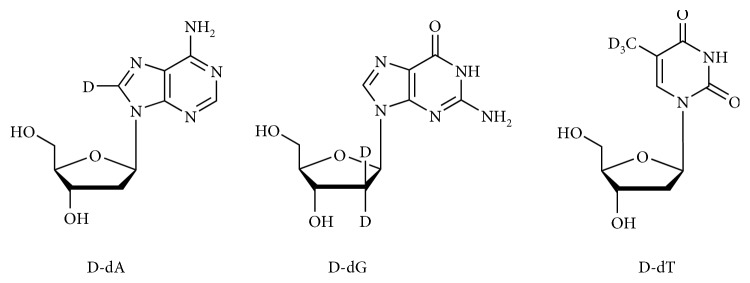


**Figure 3:**
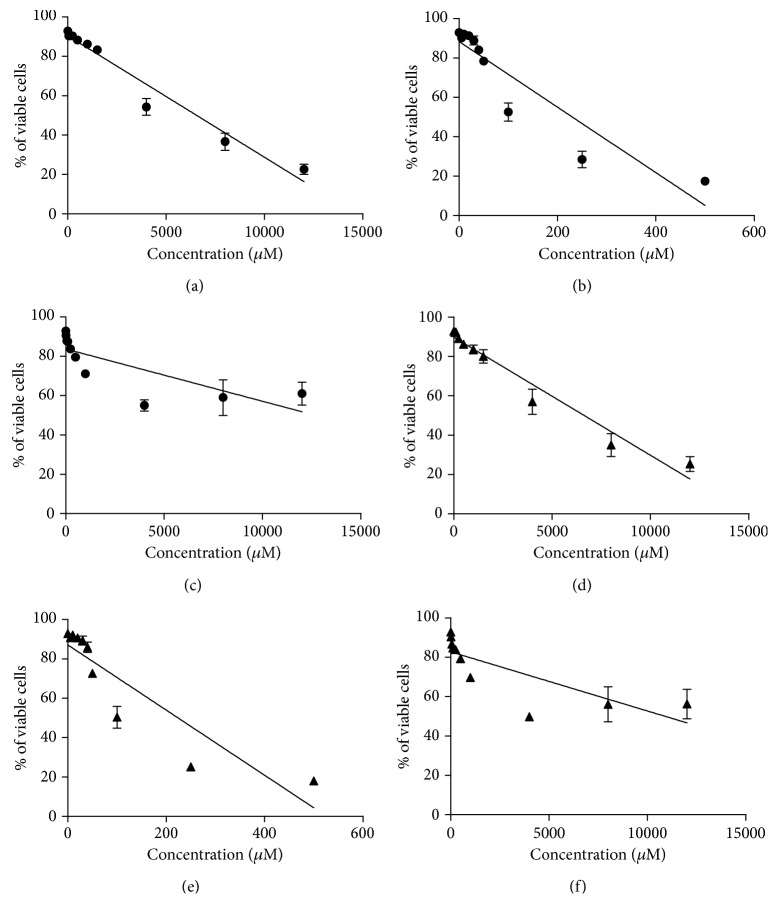
Effects on cell viability after 24 h of treatment of Jurkat cells with D-dA (a), D-dG (b), D-dT (c), dA (d), dG (e), or dT (f). Cells were stained with 7-AAD, and viability was determined by flow cytometry. We show the results as mean ± SEM of at least three experiments. Linear regression lines are shown.

**Figure 4:**
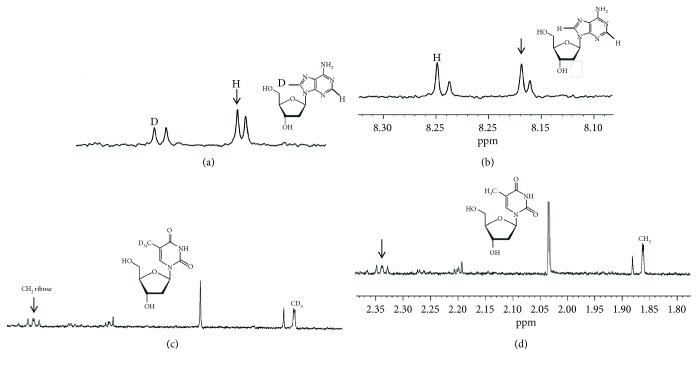
^1^H NMR spectra region between 8.10 and 8.35 ppm. (a) The digested DNA from the cells treated with D-dA. (b) The digested DNA from the cells treated with dA. ^1^H NMR spectra region between 1.80 and 2.35 ppm. (c) The digested DNA from the cells treated with D-dT. (d) The digested DNA from the cells treated with dT.

It is well known that reactive hydroxyl radical (^•^OH) can react with DNA by addition to double bonds of DNA bases or by abstraction of an H atom from the methyl group of thymine and each of the C-H bonds of 2-deoxyribose [31]. Thus, representative D-nucleosides, containing deuterons in different positions were selected, namely, D-dA, D-dG, and D-dT (see Scheme 1). In order to accelerate the incorporation of D compounds into cells, cells displaying a robust proliferation rate, namely, Jurkat cells, were used. Jurkat cells were incubated 24 h with each of the D-nucleosides to allow their incorporation into genomic DNA. NMR spectrometry was preliminary performed to show that D-nucleosides were efficiently incorporated into the Jurkat DNA. Concentrations of D-nucleosides or nucleosides devoid of any cytostatic/cytotoxic effect (Figure 3) were used, i.e., up to 500 *μ*M for D-dA, up to 50 *μ*M for D-dG, and up to 100 *μ*M for D-dT. In Figure 4, we show two different ^1^H NMR spectra regions: left panel (8.10–8.30 ppm) for the dA signals and right panel (1.80–2.35 ppm) for the deoxythymidine (dT) signals. We acquired two different samples for the dA: sample a—digested DNA from cells treated with D-dA (Figure 4(a)), and sample b—digested DNA from cells treated with dA (Figure 4(b)). We observed 4 different signals coming from dA. The dA was characterized by two H signals at 8.25 and 8.17 ppm, respectively. The two resonances are visible when we added dA in the samples (as indicated in the spectrum sample b). To prove that D-dA was incorporated into the Jurkat DNA chain, we prepared a sample where D-dA was added. In the spectrum of this last analysis (sample a), we clearly observed that the proton signal at 8.17 ppm shows the same intensity, while the deuterated signal at 8.25 ppm was lower compared to spectrum sample b. We acquired also two different samples for the dT: sample c—digested DNA from cells treated with D-dT (Figure 4(c)), and sample d—digested DNA from cells treated with dT (Figure 4(d)). The dT was characterized principally by two signals at 1.86 ppm (CH_3_) and 2.34 ppm (CH_2_ ribose) as shown in Figure 4. To prove that D-dT was incorporated into Jurkat DNA chain, we prepared a sample where D-dT was added. In the spectrum of this last analysis (sample c), we clearly observed that the proton signal at 2.34 ppm shows the same intensity, while the deuterated signal at 1.86 ppm was lower compared to spectrum sample d. These findings are also supported by the calculation of the peak area coming from the different signals at 8.25 and 8.17 ppm for dA and at 2.36 and 1.86 ppm for dT, showing an incorporation of dA of about 30-40% and of dT of about 10-20%.

Jurkat cells with normal (control) or D-enriched DNA (D-DNA Jurkat) were exposed to H_2_O_2_, the most representative oxidative stressing and DNA damaging agent [32]. This agent causes oxidative DNA lesions, which are mostly converted into and detected as DNA single-strand breaks, whose level was determined using FHA or, in selected experiments, the comet assay. Appropriate sham-treated samples, incubated 24 h with the same concentrations of the corresponding non-D-nucleosides, were always included in the experiments described below; no statistically significant difference was found between these samples and the control Jurkat cells (incubated with no supplemental nucleoside).

Last but not least, to see whether longer DNA-preincubation times with the same D-nucleosides concentrations could lead to a more efficient D incorporation and consequently to different protective effects, some experiments have been run with cells prelabelled for 36 or 48 h (i.e., 2-3 cell cycles). Under these conditions, the protective effects of D enrichment were not significantly different as compared to 24 h prelabelling (data not shown) suggesting that 1-2 days of preincubations resulted in a similarly efficient incorporation of D-nucleosides into nuclear DNA. However, although it is beyond the scope of the present study, it will be interesting to determine the effect of longer incubations with lower D-nucleosides concentrations on oxidative DNA damage (for example, 1 month with one tenth of the D-nucleotides concentrations used herein, which would result in the progressive D enrichment of ≥50 cell generations). That setting should be more representative of a chronic situation allowing a more detailed picture of the dose and time relationships of D incorporation on DNA sensitivity to oxidant injury.

**Figure 5:**
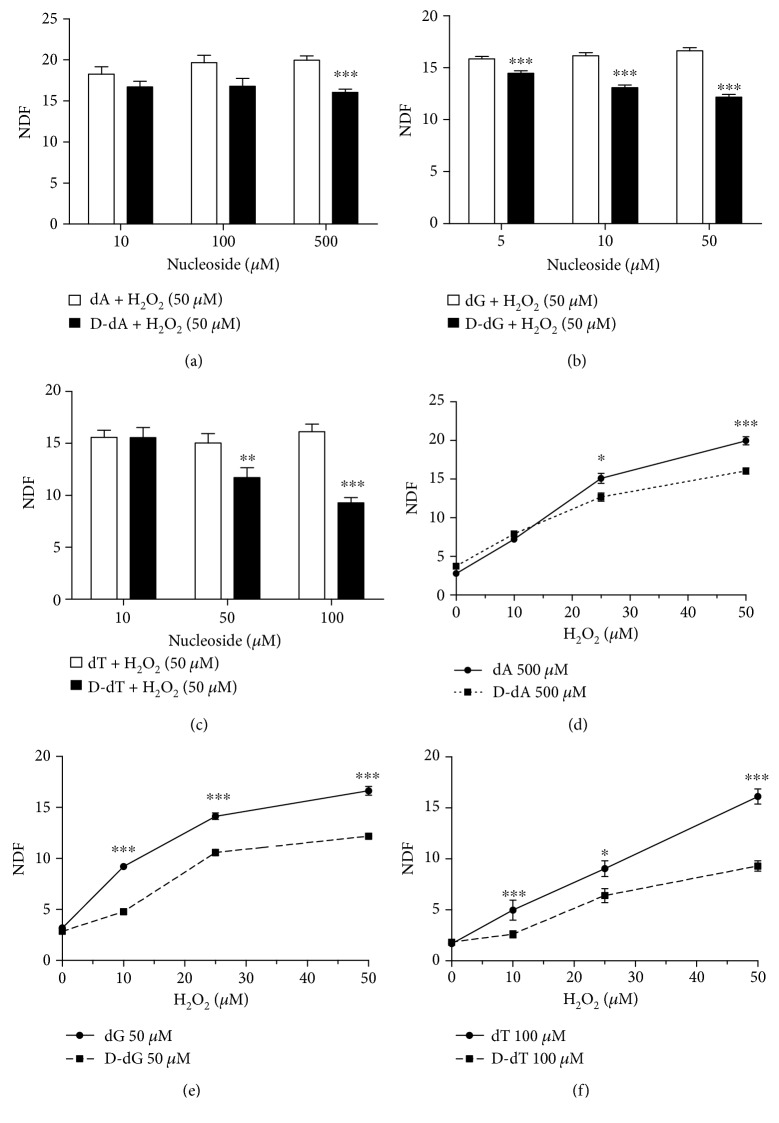
Effect of deuterated nucleoside incorporation on the extent of oxidative DNA damage in Jurkat cells. (a–c) The effect of increasing concentrations of D-dA, D-dG, and D-dT, respectively, on the extent of DNA single-strand breakage caused by a 30 min challenge with 50 *μ*M H_2_O_2_. (d–f) The effect of a fixed dose of D-dA (500 *μ*M), D-dG (50 *μ*M), and D-dT (100 *μ*M), respectively, on the DNA damage induced by increasing concentrations of H_2_O_2_. NDF (nuclear diffusion factor) represents the extent of DNA damage measured by FHA. We show the results as mean ± SEM of at least five experiments. ^∗^*P* < 0.05, ^∗∗^*P* < 0.01, and ^∗∗∗^*P* < 0.001 (one-way ANOVA).

Incorporation of D-nucleosides caused a statistically significant reduction of the extent of DNA damage caused by a single H_2_O_2_ concentration (50 *μ*M for 30 min at 37°C), at least at one of the concentrations tested (Figures 5(a)–5(c)). The order of potency (i.e., the D-nucleoside producing the higher reduction at the lower concentration) was D-dG > D-dT > D-dA. The highest degree of reduction was observed with 100 *μ*M D-dT. The effect of a fixed (the most active) dose of each of the D-nucleosides on the extent of DNA damage caused by increasing H_2_O_2_ concentrations was also tested and confirmed the ability of D-nucleosides to reduce the sensitivity of nuclear DNA to oxidative damage (Figures 5(d)–5(f)).

The rank order of potency (D-dG > D-dT > D-dA) seems to reflect the importance of D isotope effect in the hydrogen atom abstraction reaction. Actually, with D-dT and D-dG, ^•^OH radicals are expected to abstract a D-atom from the methyl group of thymine and C-D bonds of 2-deoxyribose, respectively. D-atom bound at C8 position in adenosine is instead not extractable, and the substitution of H with D in this position is expected to have a smaller effect. The susceptibility of the nucleosides to oxidative damage might represent an additional factor. Indeed, the higher the susceptibility of the non-D-nucleosides, the higher the expected protective effect related to D density. In this light, it is not surprising that the higher potency was associated with D-dG, since guanosine is known to be the most frequent target of oxidative damage.

### 3.2. D-Nucleosides Do Not Affect DNA Repair

**Figure 6:**
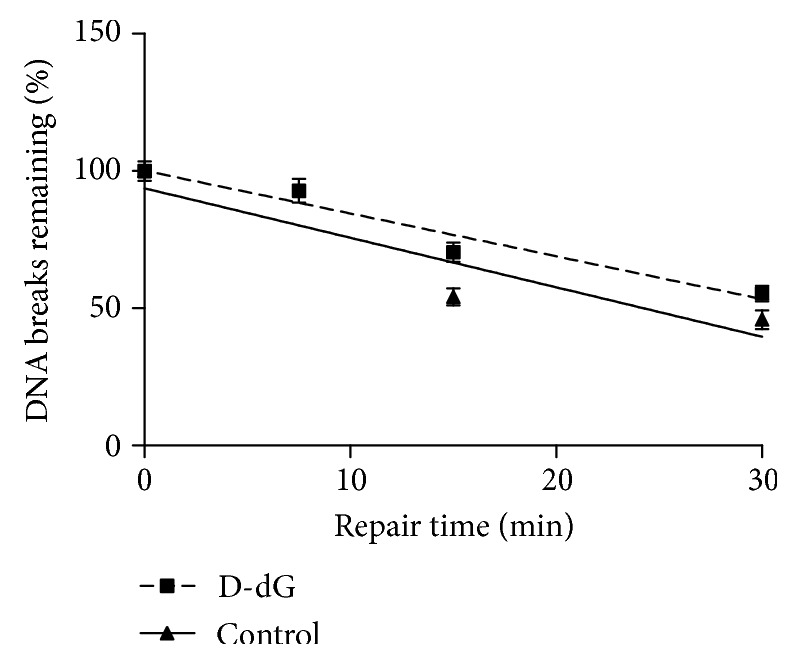
The effect of D-dG incorporation on the repair rate of DNA single-strand breaks induced by hydrogen peroxide in Jurkat cells. Control or D-dG pretreated cells were incubated for 30 min with 50 *μ*M H_2_O_2_ and then allowed to repair. DNA breaks were measured either immediately after treatment or after different times of repair. Each point is the mean of experimental values obtained in five separate determinations. Linear regression lines are shown.

D-DNA Jurkat were exposed to H_2_O_2_ (50 *μ*M for 30 min) and then allowed to repair DNA breaks in oxidant-free medium: analysis of the repair kinetics (Figure 6 shows representative results obtained with control and D-dG) found that they were superimposable in the control or D-DNA cells, with slopes values of −1.433 ± 0.2424 (control), −1.35 ± 0.17 (D-dT 100 *μ*M), −1.49 ± 0.14 (D-dG 50 *μ*M), and −1.35 ± 0.24 (D-dA 500 *μ*M). The mean *T*_1/2_ (i.e., the time required to reduce the initial level of breaks by 50%) of the lesions caused in D-DNA Jurkat and control Jurkat was 34.4 ± 0.61 min. This finding clearly shows that genomic D-nucleotides incorporated into nuclear DNA, in virtue of their subtle chemical differences as compared to nondeuterated ones, do not affect the activity or the efficiency of the DNA repair machinery in removing oxidative DNA breaks. Similarly, prolonging the D-labelling times to 36 or 48 h did not affect the repair kinetics of oxidatively damaged Jurkat cells' nuclear DNA (data not shown).

### 3.3. D-AAs Do Not Affect Protein Synthesis in Cell-Free Systems

**Scheme 2:**
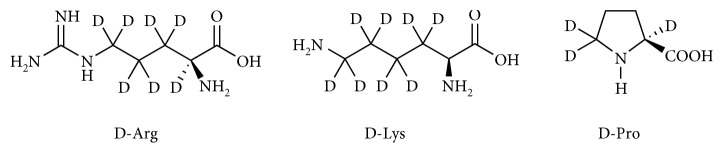


**Figure 7:**
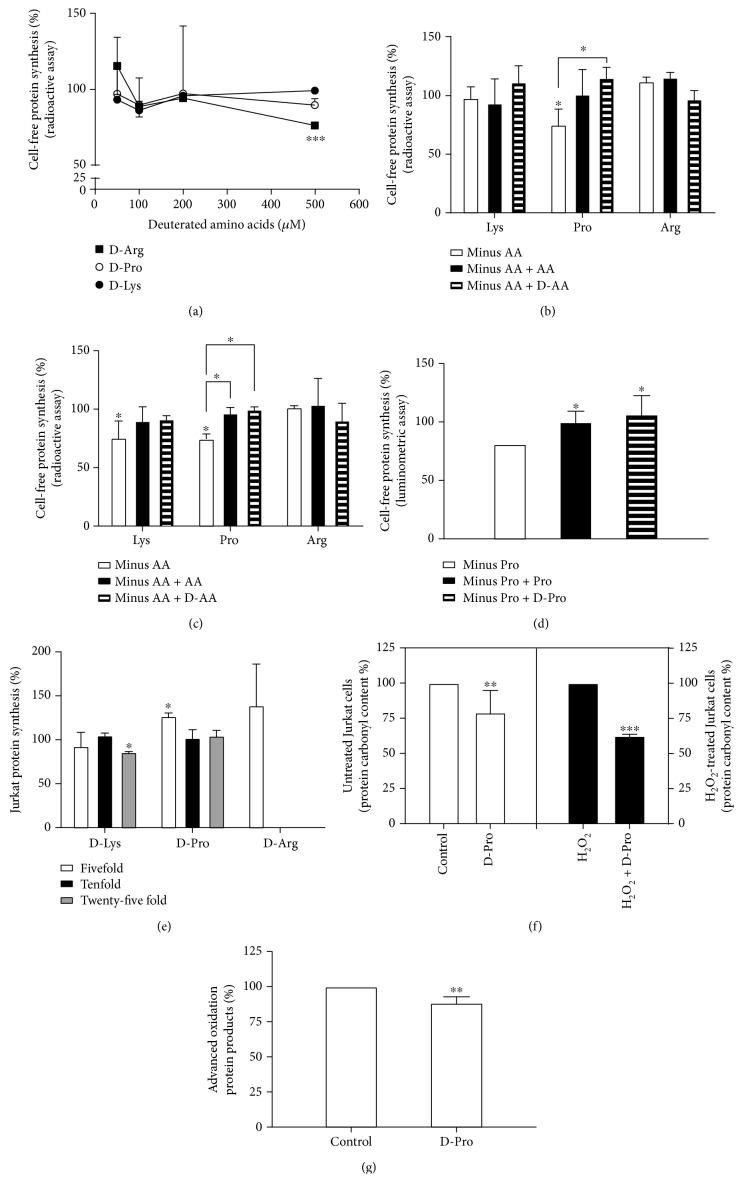
Effects of D-AA on protein synthesis and damage. (a) Protein synthesis by rabbit reticulocyte lysate translating endogenous messengers measured by a radioactive assay in the presence of D-AA at the concentration indicated in the figure. The extent of translation was measured as the incorporation into proteins of [^3^H]leucine and expressed as percentage activity. (b) Protein synthesis (as described in panel (a)) performed in the absence of the indicated AA (minus AA), after the addition of the corresponding AA (minus AA + AA), or substituting the deuterated form (50 *μ*M) for the indicated AA (minus AA + D-AA). (c) Protein synthesis performed (as in panel (b)) with gel-filtered rabbit reticulocyte lysate. (d) Protein synthesis by rabbit reticulocyte lysate translating the mRNA encoding *Renilla reniformis* luciferase measured by a luminometric assay in the absence and in the presence of added Pro or substituting D-Pro (50 *μ*M) for Pro. The extent of translation was measured as the emission of luminescence and expressed as percentage activity. (e) Protein synthesis in Jurkat cells preincubated with D-AA. The rate of translation was measured as the incorporation into proteins of [^3^H]leucine and expressed as percentage activity. (f) Protein carbonyl formation in Jurkat cells preincubated for 20 h in the absence (control) or in the presence of D-Pro and subsequently untreated or treated with hydrogen peroxide. The carbonyl levels were 1.8 and 2.7 nmol/mg of protein in the control untreated cells or control H_2_O_2_-treated cells, respectively. (g) Advanced oxidation protein products in Jurkat cells preincubated for 20 h in the absence (control) or in the presence of D-Pro (see Materials and Methods). The level of advanced oxidation protein product in the control cells was 0.8 nmol/mg of protein. ^∗^*P* < 0.05, ^∗∗^*P* < 0.01, and ^∗∗∗^*P* < 0.001.

Several amino acids (Arg, Lys, and Pro) are quite sensitive to oxidative damage in whole proteins forming carbonyl derivatives in their side chains [27]. We tested also the protective effect of some representative D-AAs having D in their side chains (see Scheme 2) on protein oxidation. Also in this case, H/D substitutions concern positions directly involved in the oxidative damage of proteins [10]. First, we studied their effect on protein synthesis in cell-free systems. Cell-free translation assays, such as the unfractionated rabbit reticulocyte lysate, allow measuring the effect on protein synthesis of a given compound under stringent conditions. This quite efficient cell-free system closely reproduces the translation of natural messengers into proteins taking advantage of a very short incubation time that renders unlikely biotransformation or degradation of the studied compounds [20]. As shown in Figure 7(a), the rate of translation did not change in the presence of D-AA supplementation, with the exception of D-Arg, which induced a slight inhibition (~25%) at the highest concentration tested (corresponding to tenfold excess with respect to the added normal AA).  Figure 7(b) shows the results obtained in omitting one AA from the reaction mixture in the absence and in the presence of the corresponding AA or D-AA. Depletion of Arg and Lys did not reduce protein synthesis, and the addition of the corresponding AA did not change the pattern. This is in keeping with the notion that the pools of Arg and Lys are very high in rabbit reticulocyte lysate [22]. Addition of the D-form caused a nonsignificant inhibition (D-Arg) or stimulation (D-Lys). Conversely, avoiding the addition of Pro in the reaction mixtures impaired translation, which was resumed and even stimulated in the presence of Pro or D-Pro. Gel filtration of the lysate to remove the excess of naturally occurring AAs did not substantially modify the results (Figure 7(c)). Thus, the D-form of Pro can be used in cell-free systems without altering the efficiency of protein synthesis measured as the rate of incorporation into protein of a radioactive AA (Figures 7(a)–7(c)). It should be noted that this technique only measures the progress in translation, without giving any information on the status of the synthesized protein. The measurement of the fidelity of translation is mandatory to assess the functional activity of the translated proteins. Gel-filtered rabbit reticulocyte lysate was challenged with a capped mRNA encoding *R. reniformis* luciferase. After translation into protein (90 min at 37°C), the enzymatic reaction catalyzed by the 37 kDa luciferase was quantified by a luminometer. A luminescent signal can be recorded only in the presence of a full-translated active enzyme. Luciferase activity was completely recovered after incorporation into proteins of D-Pro and did not change with respect to control (Figure 7(d)); besides, it is well known that mutations at Pro 220 or Pro 224/286 in the active site are associated with >99% or 90-99% impairment of the enzymatic activity of the luciferase [33]. Thus, D-Pro was considered a good candidate to assess a protective effect on protein oxidation in cells.

### 3.4. D-AAs Are Well Tolerated by Cells

Jurkat cells were cultured for 20 h in complete medium (RPMI 1640 containing Arg 1.15 mM, Lys 0.22 mM, and Pro 0.174 mM) in the absence and in the presence of 5- or 10- or 25-molar excess of D-Lys and D-Pro (maximum tested concentrations equal to 5.5 or 4.35 mM, respectively). D-Arg was added only at fivefold excess to reach a maximum assayed concentration (5.75 mM) similar to that used for D-Lys and D-Pro and to avoid adverse effects on pH at higher concentrations. Cytofluorimetric determination of viable (7-AAD/annexin V-negative), apoptotic (7-AAD-negative/annexin V-positive), and necrotic (7-AAD-positive/annexin V-positive) cells did not show any difference between controls and treated cells (Table 1). The rate of protein synthesis in cells cultured as described above was measured during 30 min incubation with radioactive leucine [23]. As shown in Figure 7(e), the presence of D-Lys at the highest concentration caused a slight inhibition of translation (~15%), whereas D-Pro induced stimulation (lower concentration) or allowed full translational activity at the higher concentrations tested, hence confirming that the presence of this D-AA was well tolerated by cells. The presence of intracellular pools of free D-Pro and D-Lys (Figure 1) was demonstrated by NMR by collecting supernatants after TCA 20% precipitation of proteins from postmitochondrial supernatants of cells incubated with 5 mM (~25-fold molar excess) D-AA. The precipitated proteins were hydrolysed (2N NaOH for 24 h at 100°C), and the alkaline treatment yielded the two D-AAs which were detected by the same technique (Figure 2), thus demonstrating their incorporation into cellular proteins. Accordingly, the amount of D-Pro incorporated in cellular proteins was 39.7 nmol/mg of cellular protein.

### 3.5. D-Pro Reduces Oxidative Damage in Cellular Proteins

The protective role of D-Pro (5 mM) on cellular proteins was evaluated both on spontaneous (20 h incubation) or H_2_O_2_-induced (5 mM for 2 h) oxidative damage. Not surprisingly, as compared to DNA—the most sensitive macromolecule to oxidative attack [34]—protein damage could be observed at higher H_2_O_2_ concentrations. The incorporation into proteins of this D-AA reduced the carbonyl group contents upon spontaneous or hydrogen peroxide-induced oxidative damage (Figure 7(f)). Consistently, the spontaneous (20 h incubation) advanced oxidation protein products were significantly reduced (Figure 7(g)).

## 4. Conclusion

Due to its higher mass, D is known to exhibit different bond cleavage rates as compared to H. Based on this notion, it was shown that the substitution of D for H in oxidation-sensitive sites of target and relevant biological macromolecules under Fenton reaction-oxidative attack results in a higher resistance to oxidation itself, with no modification to their biological identity and function [35]. Using an experimental approach based on the enrichment of cellular DNA and proteins with deuterated precursors, we have demonstrated for the first time in a living and proliferating cell system that the presence of increasing proportions of deuterated nucleosides and amino acids renders these macromolecules and the cells themselves more resistant to oxidative damage caused by H_2_O_2_, a very common toxicant generated within both intra and extracellular milieu as a result of cellular biochemistry, UV and visible light, X irradiation, and redox reactions [34]. As suggested by Cooper et al. [36], our results also show that isotopic effect may influence the product stability under otherwise identical reaction conditions.

The D-associated protective effect against oxidative damage observed in the present study in DNA and proteins of living Jurkat cells is clearly dependent on a very unnatural D enrichment in biomolecules, far higher as compared to that due to naturally occurring random H/D exchange and the quantity of D in contemporary nature (0.015%) [37]: under these conditions, it is unlikely that the D protective effects reported here can be observed, due to the very slight amount of D in macromolecules. However, over a billion years' time-scale (Earth life arose around 3.5 billions of years ago), it is conceivable that the abundance of D above the overall cosmic level in Earth's biosphere may have had a positive impact on the emergence, propagation, and evolution of life by stabilizing critical biomolecules against common types of damages, such as those held by oxidants. Notably, Somlyai et al. [38], although using a completely different experimental approach based on growing cells in D-depleted water, showed that D positively affects cell growth demonstrating an unknown biological relevance of D.

Our findings point to a new, subtle but likely role of D in the protection of critical biomolecules where its inclusion might have facilitated their resistance during the infinite generations of life entities, cells, and organisms which took place over a 3.5-billion-year history of terrestrial life. In this light, D abundance in Earth's biosphere might represent another tile in the complex scenario of the planet's tight requirements [39], i.e., the planetary habitable zone conditions, for life to arise and survive identified so far.

## Figures and Tables

**Table 1:** Viability of Jurkat cells preincubated (24 h) with D-AA.

D-AA	7-AAD/annexin V-negative cells (%)
None	92.3 ± 0.6
D-Arg	
Fivefold∗	90.5 ± 0.7
D-Lys	
Fivefold	92.3 ± 1.5
Tenfold	92.7 ± 0.6
25-fold	92.0 ± 1.0
D-Pro	
Fivefold	91.3 ± 0.6
Tenfold	91.3 ± 0.6
25-fold	91.0 ± 1.0

∗Fold excess with respect to normal added AA.

## Data Availability

The data used to support the findings of this study are available from the corresponding author upon request.

## References

[1] Matteucci F. (2000). The cosmic origin of deuterium. *Nature*.

[2] Hartogh P., Lis D. C., Bockelée-Morvan D. (2011). Ocean-like water in the Jupiter-family comet 103P/Hartley 2. *Nature*.

[3] Charnley S. B., Rodgers S. D., Kuan Y.-J., Huang H.-C. (2002). Biomolecules in the interstellar medium and in comets. *Advances in Space Research*.

[4] Millar T. J. (2005). Deuterium in interstellar clouds. *Astronomy & Geophysics*.

[5] Harman D. (1980). Free radical theory of aging: origin of life, evolution, and aging. *Age*.

[6] van Loon B., Markkanen E., Hubscher U. (2010). Oxygen as a friend and enemy: how to combat the mutational potential of 8-oxo-guanine. *DNA Repair (Amst)*.

[7] Cathcart R., Schwiers E., Saul R. L., Ames B. N. (1984). Thymine glycol and thymidine glycol in human and rat urine: a possible assay for oxidative DNA damage. *Proceedings of the National Academy of Sciences of the United States of America*.

[8] Huang H., Imoto S., Greenberg M. M. (2009). The Mutagenicity of Thymidine Glycol inEscherichia coliIs Increased When It Is Part of a Tandem Lesion. *Biochemistry*.

[9] Lentner C. (1984). Physical chemistry, composition of blood, haematology, somatometric data. *Geigy Scientific Tables*.

[10] Davies K. J. (1987). Protein damage and degradation by oxygen radicals. I. General aspects. *The Journal of Biological Chemistry*.

[11] Simmons E. M., Hartwig J. F. (2012). On the interpretation of deuterium kinetic isotope effects in C-H bond functionalizations by transition-metal complexes. *Angewandte Chemie (International Ed. in English)*.

[12] Turrini E., Calcabrini C., Tacchini M. (2018). In vitro study of the cytotoxic, cytostatic, and antigenotoxic profile of Hemidesmus indicus (L.) R.Br. (Apocynaceae) crude drug extract on T lymphoblastic cells. *Toxins*.

[13] Turrini E., Calcabrini C., Sestili P. (2016). Withania somnifera induces cytotoxic and cytostatic effects on human T leukemia cells. *Toxins*.

[14] Quinlivan E. P., Gregory J. F. (2008). DNA methylation determination by liquid chromatography-tandem mass spectrometry using novel biosynthetic [U-15N]deoxycytidine and [U-15N]methyldeoxycytidine internal standards. *Nucleic Acids Research*.

[15] Price W. S., Hayamizu K., Ide H., Arata Y. (1999). Strategies for diagnosing and alleviating artifactual attenuation associated with large gradient pulses in PGSE NMR diffusion measurements. *Journal of Magnetic Resonance*.

[16] Sestili P., Martinelli C., Stocchi V. (2006). The fast halo assay: an improved method to quantify genomic DNA strand breakage at the single-cell level. *Mutation Research*.

[17] Sestili P., Fimognari C. (2014). Alkaline nuclear dispersion assays for the determination of DNA damage at the single cell level. *Methods in Molecular Biology*.

[18] Tommasini I., Sestili P., Cantoni O. (2002). Delayed formation of hydrogen peroxide mediates the lethal response evoked by peroxynitrite in U937 cells. *Molecular Pharmacology*.

[19] Sestili P., Guidarelli A., Dacha M., Cantoni O. (1998). Quercetin prevents DNA single strand breakage and cytotoxicity caused by tert-butylhydroperoxide: free radical scavenging versus iron chelating mechanism. *Free Radical Biology & Medicine*.

[20] Penzo M., Carnicelli D., Montanaro L., Brigotti M. (2016). A reconstituted cell-free assay for the evaluation of the intrinsic activity of purified human ribosomes. *Nature Protocols*.

[21] Brigotti M., Petronini P. G., Carnicelli D. (2003). Effects of osmolarity, ions and compatible osmolytes on cell-free protein synthesis. *The Biochemical Journal*.

[22] Jackson R. J., Hunt T. (1983). [4] Preparation and use of nuclease-treated rabbit reticulocyte lysates for the translation of eukaryotic messenger RNA. *Methods in Enzymology*.

[23] Petronini P. G., Tramacere M., Mazzini A., Piedimonte G., Silvotti L., Borghetti A. F. (1987). Hyperosmolarity-induced stress proteins in chick embryo fibroblasts. *Experimental Cell Research*.

[24] Warner R. C. (1942). The alkaline hydrolysis of egg albumin. *The Journal of Biological Chemistry*.

[25] Bradford M. M. (1976). A rapid and sensitive method for the quantitation of microgram quantities of protein utilizing the principle of protein-dye binding. *Analytical Biochemistry*.

[26] Kim Y. H., Lee Y. S., Choi E. M. (2011). Linarin isolated from Buddleja officinalis prevents hydrogen peroxide-induced dysfunction in osteoblastic MC3T3-E1 cells. *Cellular Immunology*.

[27] Reznick A. Z., Packer L. (1994). [38] Oxidative damage to proteins: Spectrophotometric method for carbonyl assay. *Methods in Enzymology*.

[28] Levine R. L., Garland D., Oliver C. N. (1990). [49] Determination of carbonyl content in oxidatively modified proteins. *Methods in Enzymology*.

[29] Luo S., Wehr N. B. (2009). Protein carbonylation: avoiding pitfalls in the 2, 4-dinitrophenylhydrazine assay. *Redox Report*.

[30] Kayali R., Cakatay U., Akcay T., Altug T. (2006). Effect of alpha-lipoic acid supplementation on markers of protein oxidation in post-mitotic tissues of ageing rat. *Cell Biochemistry and Function*.

[31] von Sonntag C. (1987). *The Chemical Basis of Radiation Biology*.

[32] Imlay J. A. (2008). Cellular defenses against superoxide and hydrogen peroxide. *Annual Review of Biochemistry*.

[33] Loening A. M., Wu A. M., Gambhir S. S. (2007). Red-shifted Renilla reniformis luciferase variants for imaging in living subjects. *Nature Methods*.

[34] Sestili P., Piedimonte G., Cattabeni F., Cantoni O. (1986). Induction of DNA breakage and suppression of DNA synthesis by the OH radical generated in a Fenton-like reaction. *Biochemistry International*.

[35] Hill S., Lamberson C. R., Xu L. (2012). Small amounts of isotope-reinforced polyunsaturated fatty acids suppress lipid autoxidation. *Free Radical Biology & Medicine*.

[36] Cooper G. J. T., Surman A. J., McIver J. (2017). Miller-Urey spark-discharge experiments in the deuterium world. *Angewandte Chemie (International Ed. in English)*.

[37] Mosin O. (2008). Heavy water, Molecular Evolution And Life On Our Planet. *Science 2.0*.

[38] Somlyai G., Jancso G., Jakli G. (1993). Naturally occurring deuterium is essential for the normal growth rate of cells. *FEBS Letters*.

[39] Seager S. (2013). Exoplanet habitability. *Science*.

